# Monitoring Intervention Coverage in the Context of Universal Health Coverage

**DOI:** 10.1371/journal.pmed.1001728

**Published:** 2014-09-22

**Authors:** Ties Boerma, Carla AbouZahr, David Evans, Tim Evans

**Affiliations:** 1 World Health Organization, Geneva, Switzerland; 2 Independent consultant; 3 World Bank Group, Washington (D.C.), United States of America

## Abstract

Monitoring universal health coverage (UHC) focuses on information on health intervention coverage and financial protection. This paper addresses monitoring intervention coverage, related to the full spectrum of UHC, including health promotion and disease prevention, treatment, rehabilitation, and palliation. A comprehensive core set of indicators most relevant to the country situation should be monitored on a regular basis as part of health progress and systems performance assessment for all countries. UHC monitoring should be embedded in a broad results framework for the country health system, but focus on indicators related to the coverage of interventions that most directly reflect the results of UHC investments and strategies in each country. A set of tracer coverage indicators can be selected, divided into two groups—promotion/prevention, and treatment/care—as illustrated in this paper. Disaggregation of the indicators by the main equity stratifiers is critical to monitor progress in all population groups. Targets need to be set in accordance with baselines, historical rate of progress, and measurement considerations. Critical measurement gaps also exist, especially for treatment indicators, covering issues such as mental health, injuries, chronic conditions, surgical interventions, rehabilitation, and palliation. Consequently, further research and proxy indicators need to be used in the interim. Ideally, indicators should include a quality of intervention dimension. For some interventions, use of a single indicator is feasible, such as management of hypertension; but in many areas additional indicators are needed to capture quality of service provision. The monitoring of UHC has significant implications for health information systems. Major data gaps will need to be filled. At a minimum, countries will need to administer regular household health surveys with biological and clinical data collection. Countries will also need to improve the production of reliable, comprehensive, and timely health facility data.

*Please see later in the article for the Editors' Summary*

## Abstract 2

As part of the Universal Health Coverage Collection, Ties Boerma and colleagues discuss monitoring intervention coverage related to the full spectrum of UHC, including health promotion and disease prevention, treatment, rehabilitation, and palliation.

*Please see later in the article for the Editors' Summary*

**Summary Points**

- Monitoring universal health coverage (UHC) should be integral to overall tracking of health progress and performance, which requires regular assessment of health system inputs (finances, health workforce, and medicines), outputs (service provision), coverage of interventions, and health impacts, as well as the social determinants of health.
- Within this overall context, we propose that UHC monitoring focus on financial protection and intervention coverage indicators, with a strong equity focus. This paper focuses on intervention coverage.
- Progress towards UHC should be tracked using tracer intervention coverage indicators selected on the basis of objective considerations and designed to keep the numbers of indicators small and manageable while covering a range of health interventions to capture the essence of the UHC goal.
- Since UHC is about progressive realization and countries differ in epidemiology, health systems, socioeconomic development, and people's expectations, the indicator sets will not be the same everywhere.
- Coverage indicators should cover promotion and prevention, as well as treatment, rehabilitation, and palliation. While there are several suitable indicators for the first two, there are major gaps for coverage indicators of treatment, as population need for treatment is difficult to measure.
- A small set of well-established international intervention tracer coverage indicators can be identified for monitoring UHC. Where no good indicators are currently available, proxy indicators and equity analysis of service utilization can provide some insights.
- Special attention needs to be paid to quality of services, either through the tracer indicator itself (referred to as effective coverage) or through additional indicators on quality of services or health impact of the intervention.
- Targets should be set in accordance with baseline, historical rate of progress, and measurement considerations.
- The main data sources of intervention coverage indicators are household surveys and health facility reports. Investments in both are needed to improve the ability of countries to monitor progress towards UHC.
- It is essential to find effective ways of communicating progress towards UHC in ways that are meaningful to the general public and that capture the attention of policy makers.

This paper is part of the PLOS Universal Health Coverage Collection.

## Introduction

Universal health coverage (UHC) has been defined as the ability of all people who need health services to receive them without incurring financial hardship [1]. UHC consists of two inter-related components: coverage with health services, including promotion, prevention, treatment, rehabilitation, and palliation, and coverage with financial protection, for everyone. The former captures the aspiration that all people obtain the health services they need, while the latter aims to ensure that they do not suffer financial hardship linked to paying for these services.

For all countries, moving towards UHC is a process of progressive realization. It is about making progress on several fronts: the available range of services; the quality of the services; the proportion of the costs of those services covered; and the proportion of the population covered. For richer countries, the main challenges relate to protecting and extending past gains in the face of financial constraints, ageing populations, new health threats, continuous advances in technologies capable of extending life or improving health, and increasing expectations on the part of the public. For the poorest countries, the challenge is to initially ensure basic essential services to the whole population. The diversity in the nature of the challenge has implications for the selection of indicators for monitoring of progress towards UHC goals in countries.

Overall monitoring of health progress and health system performance uses a range of indicators that measure determinants of health, health sector inputs such as finances and health workforce, outputs such as access to and quality of services, coverage of interventions, and health impact. For UHC monitoring we propose a focus on the level and distribution of coverage of health interventions and financial protection [2]. These are the most direct results of country UHC strategies and investments.

This paper is part of a PLOS Collection on UHC monitoring and focuses on the measurement and monitoring of health intervention coverage in the context of UHC. Monitoring financial protection is discussed in an accompanying paper in this Collection [3]. It should be stressed, however, that UHC requires simultaneous monitoring of intervention coverage and financial protection, with an equity focus.

First, we present an overall results framework for monitoring health system performance that can be used to track progress towards UHC in countries and argue for the focus on intervention coverage indicators. The framework has been applied in different ways in several country case studies in this PLOS Collection. Subsequently we examine the current coverage indicators for UHC, from health promotion to palliative care, and discuss the suitability of a set of tracer indicators for multiple intervention areas, including a quality of care dimension. We identify the main measurement gaps and summarise the investments in health information systems that will be needed to address them.

## General Health Sector Monitoring Framework

**Figure 1:**
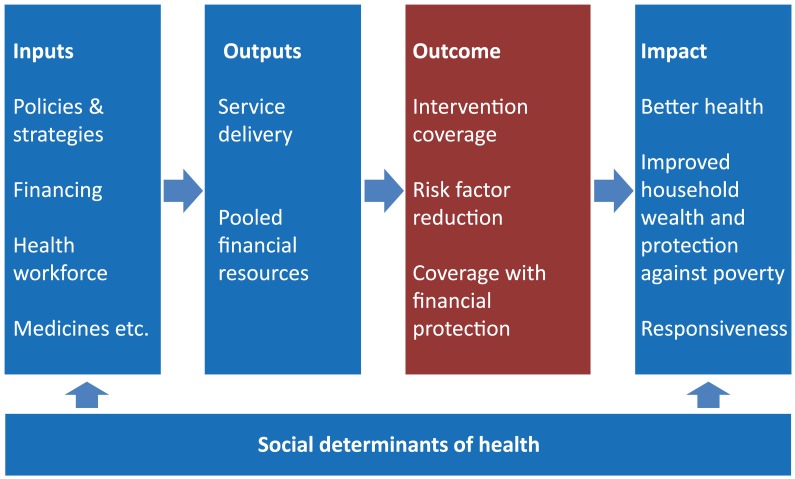
Results chain framework for monitoring health sector progress and performance: focus of UHC monitoring in the red box.

UHC monitoring constitutes one part of a broader results framework that is commonly used for monitoring and evaluation of progress and performance of specific programmes [4]–[7] and the health system [8] by many countries and globally. Figure 1 presents a results framework where health sector inputs such as money and health workforce lead to outputs such as access to and quality of services, coverage of interventions, and ultimately to health impact, that is, improved levels and distribution of health and wellbeing, and improved health systems responsiveness. Results at each step are affected by health system policies and influenced by the social determinants of health. To make progress towards the goal of UHC countries will have to advance in terms of health system inputs, outputs, and coverage of good quality services in all population groups. In this paper, we propose to focus on the coverage indicators of the results framework, i.e., people receiving the services they need, as the most direct measures of UHC progress in the population.

In addition, improved health status of the population is indicative of UHC progress although it is also influenced by socioeconomic, environmental, nutritional, and other factors. Input indicators, such as total health expenditure per capita or health workforce density, and output indicators, such as access to services (whether the health services that people might need are available, close to them), help to determine or explain observed levels of coverage with both health services and financial protection and are useful in identifying policy levers that might be used to improve coverage. However, they are conceptually different to the concept of UHC in that they are purely instrumental—they are not valued for their own sake but are ways of ensuring that people can receive interventions.

Output indicators, such as service availability and general service utilization, can provide an indication of the degree of access to services, but are less suitable for UHC monitoring than service coverage indicators, as they do not relate to a specific need for services. Moreover, setting targets for output indicators is difficult and often of limited policy value. Utilization of outpatient and inpatient services and interventions varies widely even in countries where access to services is supposedly very good.

Improvements in UHC coverage should, in principle, translate into improved health status. However, using health impact indicators, such as mortality and morbidity by age, sex, and cause to monitor UHC would be less suitable because they are insufficiently specific to UHC, being strongly affected by socioeconomic, environmental, behavioural, and other determinants of health. Changes in coverage are more responsive to programme inputs and occur more rapidly than for health impact; they are, therefore, of particular value for guiding policy and programme decisions.

Box 1 provides a summary of the terms of access, utilization, and coverage used in this paper [9]–[12].

**Box 1. Definitions of Terms**

**Service access**: the opportunity or ability for people to obtain the services they need without financial ruin. Access has three dimensions: physical accessibility, financial affordability, and socio-cultural acceptability.

**Physical accessibility**: the availability of good health services within reasonable reach of those who need them and of opening hours, appointment systems, and other aspects of service organization and delivery that allow people to obtain the services when they need them.

**Financial affordability**: a measure of people's ability to pay for services without financial hardship. It takes into account not only the price of the health services but also indirect and opportunity costs (e.g., the costs of transportation to and from facilities and of taking time away from work). Affordability is influenced by the wider health financing system and by household income.

**Acceptability**: captures people's willingness to seek services. Acceptability is low when patients perceive services to be ineffective or when social and cultural factors such as language or the age, sex, ethnicity, or religion of the health provider discourage them from seeking services.

**Intervention coverage**: people receiving the intervention or service among those who need it. It requires a fairly well-defined intervention that can be measured and precise measurement of the population need for the intervention. The target for a coverage indicator should be 100%.

**Effective coverage**: people who need health services obtain them in a timely manner and at a level of quality necessary to obtain the desired effect and potential health gains.

**Service utilization**: refers to people's use of a general or specific service, but is not related to the population who need the service. There is often no clear target. Examples of utilization indicators are outpatient visits per capita hospital admission rate and caesarean section rate in the general population without clear determinant of need.

Source: [9]–[12]

## Coverage Indicators

There are dozens of intervention coverage indicators that could be used to track UHC progress. Countries should select those indicators that are most relevant to their own situation. A systematic approach is needed to ensure the selection of an optimal set of indicators for the main health priorities and the identification of measurement gaps. This approach should also help avoid giving too much weight to intervention areas where many indicators are available and neglecting others that are more difficult to measure and monitor.

Indicators that monitor interventions and risk factor reductions can be classified in different ways: according to the type of intervention (promotion, prevention, treatment, rehabilitation, palliation), the type of condition or intervention area they address (related to the health Millennium Development Goals [MDGs], noncommunicable diseases [NCDs], injuries), the characteristics of the target population (e.g., by stage in the life course or sex), and the level of delivery of the interventions (from non-personal or population health measures to tertiary hospital services).

Table 1 presents a list of examples of indicators by intervention areas and type of intervention, divided into two major groups: promotion and prevention, and treatment services (including ambulatory and in-patient services). The coverage indicators are derived from existing listings, such as those adopted by Member States as part of World Health Assembly resolutions. Other international agreements include the coverage and risk factor indicators in the MDGs [13], the Countdown to 2015 for maternal, newborn, and child survival [14], and the action plan for monitoring NCDs [15]. The proposed UHC list is not intended to be comprehensive, but it does include the majority of the most commonly used indicators. Data availability and measurability of the indicators are highly variable.

**Table 1:** Examples of intervention coverage indicators by intervention area.

Area	Promotion and Prevention	Treatment, Rehabilitation, Palliation
Pregnancy care	ANC (4+ visits); TT vaccination	Treatment of pregnant women with positive syphilis test
Maternal/newborn care	Postnatal care for mother and newborn	Institutional delivery/skilled birth attendance
Family planning	Need for family planning satisfied	
Child vaccination	DPT3/pentavalent, PCV, measles, BCG immunization; fully vaccinated	
Treatment of child illness		Pneumonia to health facility/received antibiotics; diarrhoea with ORT/ORS
Child undernutrition	Exclusive breastfeeding; vitamin A supplementation; households with iodized salt	
Malaria control	ITN use among children/pregnant women, household ownership, indoor residual spraying	Child with fever taken to facility/confirmed cases treated with first line antimalarials
TB control		TB case detection rate, treatment success rate; HIV-TB patients receiving CPT
HIV prevention and treatment/STI	PMTCT among HIV positive women; voluntary HIV testing and counseling (general, risk populations); condom use at higher risk sex (general, risk populations)	Antiretroviral therapy; HIV and TB treatment among HIV infected persons with incident TB infection; STI appropriately diagnosed and treatment
Neglected tropical diseases (NTD)	Preventive treatment coverage among those at risk of NTD (e.g., schistosomiasis)	Treatment among those with NTD (e.g., cutane leishmaniasis, Buruli ulcer)
Epidemic prone diseases	Meningitis vaccination coverage; influenza vaccination (>60)	Treatment among those with epidemic disease
NCD	Non-use tobacco; adequate physical activity; non-obesity/overweight; non-heavy episodic use of alcohol; normal cholesterol	Hypertension treatment, diabetes treatment; preventive treatment among persons with elevated risk of severe cardiovascular events; CVD and stroke treatment; cardiac surgical interventions; cataract surgery
Cancer screening and vaccination	HPV vaccination; cervical cancer screening; mammography	Cancer treatment
Mental health		Depression treatment; severe mental disorder treatment
Surgical conditions		Hip/knee replacement, hernia, other types of surgery
Environmental health	Water supply from safe source; adequate sanitation	
	Exposure to good air quality	
	Modern fuels for indoor use	
Injuries	Helmet use; seatbelt use	Severe injury treatment
Rehabilitation		Assistive devices among persons with disabilities; rehabilitative surgical interventions; corrected refractive errors
Palliation		Use of opiates among those in need

ANC, antenatal care; CPT, co-trimoxazole preventive therapy; CVD, cardiovascular disease; DPT3, diphtheria, pertussis, tetanus; HPV, human papillomavirus; ITN, insecticide treated net; NTD, neglected tropical disease; ORS, oral rehydration salts; ORT, oral rehydration therapy; PCV, pneumococcal conjugate vaccine; PMTCT, prevention of mother to child transmission; STI, sexually transmitted infection; TB, tuberculosis; TT, tetanus toxoid.

Ideally, a small set of tracer indicators is identified to assess overall progress towards UHC. The choice of indicators should, to the extent possible, be based on objective considerations, but will involve a tradeoff between the desire to keep the numbers of indicators small and manageable and, at the same time, address a breadth of health interventions to capture the essence of the goal of UHC. Box 2 summarizes the considerations for selection of indicators (Table S1 provides an application). Since UHC goals are essentially about progressive realization, tracer indicators are likely to be added or changed as the country socioeconomic and epidemiological situation changes.

**Box 2. Considerations for the Selection of Indicators**

***Epidemiological relevance***

The indicator should measure an intervention associated with a significant proportion of the potential burden of disease. This measurement is partly captured by estimates of the current burden of disease but should also take into account the mortality and morbidity currently prevented by the intervention. For instance, measles may have a very small share of the total burden of disease in terms of mortality or other measure but this is because immunization coverage is high.

***Cost-effective intervention***

There should be an evidence base to show that the intervention is effective and feasible to deliver. As UHC is about progressive realization by countries, there may be a shift towards more costly and often less effective interventions as health services become more sophisticated and a country can afford more.

***Measurable: numerator***

Both numerator—the population receiving the intervention—and denominator—the population needing the intervention—of the coverage indicator should be well defined. In the numerator the intervention itself should be easy to define and understood. Health facilities should be able to unambiguously record and report the intervention, e.g., a vaccination or antiretroviral treatment. Respondents in health surveys should be able to correctly recall and report the event, such as a specific vaccination or a type of treatment.

***Measurable: denominator***

Denominators are easiest to measure for indicators where a whole population requires the intervention, as is the case for health promotion, e.g., adequate sanitary facilities or tobacco control, and for preventive measures, e.g., measles vaccination among children or antenatal care for pregnant women. Treatment coverage can only be computed if one knows the number of people with the condition.

***Target***

Related to the denominator is the target setting. It must be clear for all indicators that the ultimate target is 100%. In other words, contraceptive prevalence rate among married women or proportion of babies delivered by caesarean section are not coverage indicators.

***Equity***

Moving towards UHC is not only a matter of improving average levels but also about reducing disparities and improving equity. Therefore, indicator disaggregation should be possible by sex, age, household wealth/income, gender, residence (urban/rural, province, district), ethnicity, and other key stratifiers.

***Quality***

Simply receiving an intervention is not sufficient—the intervention needs to be delivered with the level of quality necessary to achieve the desired outcome.

***Comparable***

All indicators need to be measurable in a comparable way over time and across countries: both a global standard for the measurement of the indicator and universal applicability by countries are required. The best indicators are based on a trade-off between sensitivity to change, validity, and reliability.

***Easy to communicate***

Indicators must be easy to communicate to policy makers and the general public, which is challenging because achieving UHC necessitates the use of multiple indicators and composites or indexes to track progress.

***Data availability***

The two main data sources are population-based surveys and health facility data. The availability of quality comparable coverage data is an important factor affecting the selection of indicators.

***Part of international initiatives***

The extent to which indicators have been recommended (and used) in international initiatives should be considered, such as the MDGs. This adherence to international initiatives includes indicators with targets that have been accepted by countries for the monitoring of progress in the UN General Assembly or World Health Assembly resolutions.

***Parsimony***

The number of tracer indicators should be kept small.

### Promotion and Prevention Coverage Indicators

Commonly used indicators in this category include coverage of family planning services (measured by need satisfied among women of reproductive age), pregnancy and delivery care (e.g., antenatal care attendance), and immunization coverage (specific vaccines or full coverage), as well as coverage of interventions on behavioural risk factors. Indicators on safe water and sanitation should also be included, even though they are generally not the primary responsibility of the health sector, because improvement of health can be considered a primary purpose of these interventions.

Reduction of risk factors for chronic conditions and injuries includes policy measures that apply at a population level, such as tobacco control, which eventually translate into measurable changes in personal behaviours that can be expressed in terms of coverage. The indicators should measure the positive behaviour and are therefore presented as the inverse of the prevalence of the risk behaviour, e.g., non-use of tobacco among the adult population.

The denominator of the coverage indicators is relatively straightforward for most promotion and prevention indicators as the target population is usually all persons with certain age-sex characteristics, such as children under one year of age for immunization or pregnant women for antenatal care. For some indicators, such as the need for family planning satisfied, the denominator is more complicated as desire for another child, pregnancy, lactation, and exposure status will have to be taken into account [16].

The numerators of the coverage indicators are relatively straightforward for most interventions, provided there is a standardized intervention that can easily be recalled in surveys or reliably be reported through facility reporting systems. For some indicators such as immunization coverage, home-based records of child immunization are used in surveys to improve the quality of the recall data in household surveys.

Indicators of anthropometric status, e.g., the proportion of children underweight or stunted, are not included as they reflect health status rather than intervention coverage. UHC contributes to improvements in anthropometric status, which is affected by multiple factors, but the numerator of the indicator is not related to a specific intervention.

### Treatment Coverage Indicators

For most indicators of treatment coverage, the need for accurate health workers' recording or respondents' recall of the standardized intervention in facility data and surveys is the same as for preventive interventions. The main challenge is to accurately determine population need for the intervention, especially in settings where a large proportion of the population may not seek health services and problems therefore remain undiagnosed. This challenge applies to both acute and chronic conditions and similarly to conditions that require ambulatory or inpatient care. Therefore, for most indicators population-based surveys are required to estimate population need for treatment.

The need for treatment in the population can be measured through population-based surveys in three different ways: self-reports of the condition or medical diagnosis; presumptive diagnosis based on survey questions on signs and symptoms; and the collection of biological and clinical markers. A fourth approach relies on statistical modelling to estimate treatment need.

### Self-Reports

Self-reported medical diagnosis is commonly applied in surveys in high-income countries, where access to services is good. For instance, the National Health Interview surveys in the USA and Taiwan asked for a diagnosis of arthritis, diabetes, hypertension, and stroke. The method is however of limited value in detecting unmet need for treatment when people do not know they have the condition or do not report correctly [17].

Indicators of the coverage of interventions for injuries (e.g., caused by road traffic accidents) are often based on self-report of the event in a survey interview, as they require data on the number of injuries that would have required emergency care. Challenges are the quality of self-reports on severity of the injury and survivor bias. Because of these problems, monitoring of deaths caused by road traffic accidents has been proposed as a summary indicator of promotion, prevention, and treatment interventions [18].

Coverage of assistive devices or products among people living with disabilities has been proposed as an indicator [19], and need can be measured through questions on specific disabilities. Disability however has many dimensions and responses in surveys are highly dependent on the interview questions [20],[21]. Instruments can range from a few questions, such as the six questions to assess difficulties with seeing, hearing, mobility, cognition, self-care, and communication developed for censuses and surveys by the UN Statistical Commission Washington City working group (http://www.cdc.gov/nchs/washington_group.htm) to full questionnaires with comprehensive assessments of functioning. Considerable investment will be needed to obtain comparable data between or even within countries over time. Clinical markers, such as a vision test (see below), are a useful alternative for some disabling conditions.

### Symptom-Based Algorithms

Symptom-based questions, as the basis for diagnostic algorithms, have been used to obtain an estimate of the population need and coverage of interventions. For treatment of acute conditions in childhood, such as pneumonia, diarrhoeal diseases, and malaria the population need is estimated from a few questions to the mother or caretaker on signs and symptoms. A recent assessment concluded that such questions generate only crude measures of population need, but currently there are no better alternatives [22].

Algorithms have also been developed for chronic conditions in adulthood such as angina pectoris, arthritis, asthma, and depression and have been applied in surveys such as the World Health Survey, World Mental Health Survey, and the WHO Study on Adult Health and Ageing (SAGE) [23]–[26]. More evaluation is needed but initial results suggest that cross-country comparability is likely to be a problem and difficult to correct for. The use of algorithms may have more value in monitoring trends over time in the same population.

Efforts have also been made to determine the need for surgical interventions through symptom-based questions. For example, a recent survey in Sierra Leone, a low-income setting with major service gaps in surgical care, included a systematic head-to-toe verbal examination, and resulted in as many as 25% of the total 3,645 respondents reporting a surgical condition needing attention [27].

### Biological and Clinical Data Collection

Population need for treatment is best determined by biological and clinical tests in household surveys. Diabetes, hypertension, and vision problems can be detected through a serological test (e.g., HbA1c [28] or fasting blood glucose), blood pressure measurement, and a visual acuity test [29], respectively. The results can be used as markers of need for treatment for each condition. Questions will need to be included about current treatment, or in case of vision correction, the measurements should be applied with the corrective devices in place. Advances in technology permit an ever-growing number of biological and clinical markers to be collected through health examination surveys [30].

### Statistical Modelling

Estimating the need for treatment for HIV and tuberculosis relies on statistical models. In both cases, population-based and facility data are used as the main inputs into the models. For HIV, surveillance data from most at risk populations or antenatal clinic attendees and household surveys with HIV testing are the main prevalence inputs for the models [31]. For tuberculosis, notification rates from the clinics and more recently population-based prevalence surveys are the main data sources [32]. In addition, facility reports provide data on the numerator, i.e., the numbers of people receiving treatment.

The coverage of palliative care is an example of an indicator where both the numerator (the intervention that is defined as palliative care) and the denominator (the proportion of people who need palliative care) of the indicator have major measurement challenges. For the monitoring of the global noncommunicable diseases action plan it has been proposed to use morphine-equivalent consumption of strong opioid analgesics per death from cancer, obtained from administrative data on morphine consumption and estimated numbers of deaths from cancer, as a proxy indicator of access and coverage [15].

### Alternative Measures: Service Utilization

Very low service utilization rates are often indicative of poor access to health services [33], but the indicators lose their policy relevance once utilization rates rise because it is difficult to determine the optimal level of use. Service utilization rates do not relate to a specific need for services and target setting is difficult. Utilization rates vary widely even between countries where access to services is supposedly very good. For instance, the average for the 34, mostly high income, country members of the Organization for Cooperation on Economic Development (OECD) was about seven general practitioner visits per capita per year, ranging from three to 13 visits [34].

**Figure 2:**
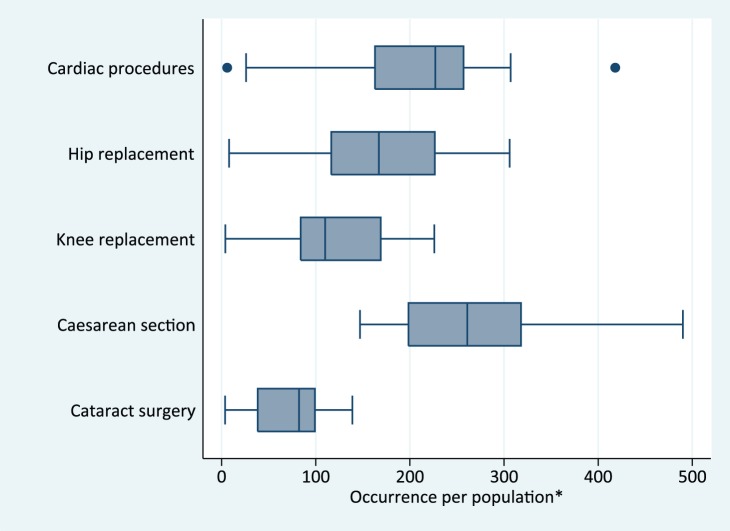
Occurrence of selected procedures by country, OECD data 34 countries. *Source: [22]. Cardiac revascularization procedures, hip replacement, knee replacement are per 100,000 population; caesarean section per 1,000 deliveries; cataract surgery per 10,000 population. The line in the box is the median, box represents the interquartile range, the whiskers the 10th and 90th percentiles.

Similarly, the need for inpatient and specialist services is difficult to measure. For instance, OECD monitoring of health care activities in mostly high-income countries focuses on indicators such as coronary revascularization procedures (e.g., angioplasty), hip replacement surgery, knee replacement surgery, caesarean section, and cataract surgery per 100,000 population [35]. Figure 2 shows the diversity of intervention rates between countries; on the basis of such variance it is difficult to come to any firm conclusions regarding coverage, i.e., what proportion of the population in need is covered.

On the other hand, in circumstances where treatment coverage is difficult to measure, disaggregating general service utilization rates by equity stratifiers offers a proxy for UHC monitoring. For example, in Chile, monitoring of hospital admission and surgical intervention rates by wealth quintiles showed that whereas admission rates were higher among the poorest quintiles compared with wealthier quintiles, the poorest had much lower surgical and specialist intervention rates compared with the wealthiest. Over time, changes in insurance payment schemes contributed to reductions in disparities in utilization rates across income groups. [35]. The problem with these measures, however, remains that the need for health services differing among population groups cannot be quantified.

One method to address this problem is to assess met or unmet need for general health services, e.g., doctor's visit or admission, through self-reports in surveys. Such questions are included in surveys such as the European Union (EU) Standards of Income and Living Conditions (SILC) surveys [36] and also in the China National Health Services survey [37]. However, these self-reports cannot be taken at face value; poorer respondents often report a lower need for health services than better-off respondents [38]. Statistical models have been developed, to assess horizontal inequity for service utilization (by wealth quintile), using adjusted need estimates derived from survey questions on self-reported health and activity limitations [39].

### Application Using Tracer Indicators

It is critical to communicate data on progress towards UHC in ways that are meaningful to the general public and that capture the attention of policy makers. One strategy is to focus on a small set of tracer indicators or a summary measure of UHC intervention coverage. Even though a summary measure will raise the debate about weights for the different components, it may nonetheless be a useful way for communicating progress towards UHC. An example of a summary index is the Countdown coverage index for maternal, newborn, and child health; this provides a summary measure of four equally weighted intervention areas based on eight indicators to monitor trends in coverage and equity within countries [40]–[42]. Results should be published both as a summary index and disaggregated to show the various components of UHC. It is noted that uncertainty ranges are not considered in the examples.

A conservative approach is to start with a small set of well-established intervention coverage indicators for which comparable data are available. Table 2 includes 12 coverage indicators: six health promotion and prevention indicators and six treatment indicators. The set includes most current MDG coverage indicators and a small set of NCD-related indicators that are part of the global action plan for monitoring the NCDs.

**Table 2:** Intervention coverage indicators with quality dimension or with additional indicators to capture service quality, and source of data.

Intervention Area	Coverage Indicator	Additional Indicators to Capture Quality of the Intervention	Data Sources for Coverage	
			Survey	Facility Data
**Promotion/prevention indicators**				
Family planning	Need for family planning satisfied among women 15–49	Quality included in indicator	++^a^	+^b^
Pregnancy care	Antenatal care: at least 4 visits (1 visit)	Type of services received	++	+
Child vaccination	Full immunization among infants	Seroconversion; disease incidence rates	++	++
NCD prevention	Non-use of tobacco among adult population (adolescents)	Quality included in the indicator	++	
Environmental health	Water: % of the population using an improved water source	Diarrhoea incidence rates; water quality	++	
	Sanitation: % of the population using an improved sanitation facility	Diarrhoea incidence rates	++	
**Treatment indicators**				
Cardiovascular disease control	Hypertension control: % of persons with hypertension who are successfully treated	Quality of care included	++	
Maternal and newborn care	Skilled birth attendance	Maternal and perinatal mortality in institutions; type of services received	++	+
Diabetes control	Diabetes control: % of persons with diabetes who are receiving successful treatment	Quality of care included	++	
TB control	TB treatment: % of cases detected and cured under DOTS	Quality included; generally presented as two indicators	+	++
HIV control	ARV therapy: % of population with advanced HIV infection using ARV	Survival rates on ARV therapy	+	++

ARV, antiretroviral; DOTS, directly observed therapy.

a ++, best data source.

b +, possible data source.

**Figure 3:**
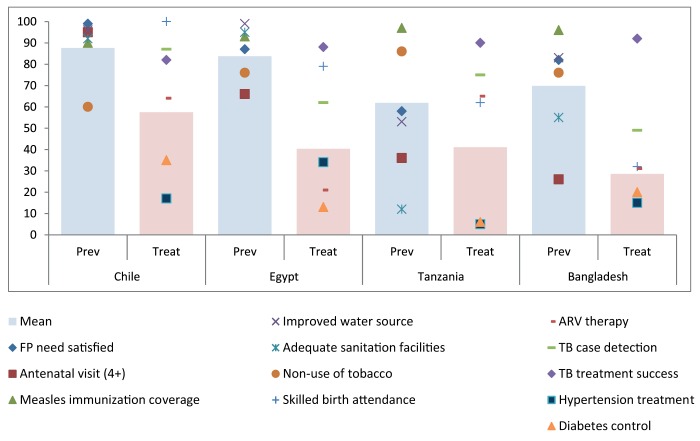
Coverage of selected indicators (dots) with mean of all intervention areas (pink bar) in selected countries.

Figure 3 provides an illustrative application of the use of 12 tracer indicators with data from four countries. Data were obtained from the WHO Global Health Observatory database [43], national household surveys such as Demographic and Health Surveys [44], and WHO STEPS surveys for risk factors [45], as well as papers that are part of this PLOS Collection [35],[46]. The means were separately computed for promotion/prevention and for treatment indicators. The two indicators for water and sanitation were given half the weight of the other four prevention/promotion indicators, as they are in the same intervention area. The treatment mean was computed as unweighted average of five interventions areas, including the single indicator areas of delivery care, HIV, hypertension, and diabetes, and TB control which included a quality dimension estimated as the product of the TB detection and TB treatment success rates (see Text S1 for computation of the mean).

In general, prevention coverage is considerably higher than for treatment (Figure 3). Chile's mean for prevention is 88% (the average is reduced due to high tobacco use), followed by Egypt (84%), Bangladesh (70%), and Tanzania (62%). Chile also has the highest mean for treatment coverage (57%), followed by Tanzania (41%), Egypt (40%), and Bangladesh (29%).

The values for effective coverage of hypertension and diabetes control were particularly low in all countries. A similar computation could be performed for the poorest income groups or other stratifiers, but this would have to exclude indicators for which no disaggregation is possible because no household survey data by wealth quintile are available (HIV and TB in this case) (Figure 3).

## Quality of Services

The aim of UHC is to provide quality services. Some indicators in Table 1 include a quality dimension and can be categorised as “effective coverage” indicators [9],[12]. Table 2 shows a quality dimension for the coverage indicators that were used the application above.

Good examples of effective coverage indicators are hypertension and diabetes treatment, which can be measured through health examination surveys. Intervention coverage is computed as individuals on treatment divided by individuals needing treatment or already on treatment. Effective coverage is computed as people with normal test results and on treatment divided by people in need of the intervention, which includes people on treatment (irrespective of test result) and people with a positive test and not on treatment. Data from WHO-supported NCD risk factor surveys in ten countries [45] show that effective coverage rates for hypertension and diabetes treatment are on average about half the coverage rates not adjusted for the success rate of treatment. This finding may be because of poor treatment adherence by the users as well as suboptimal efficacy of the treatment itself.

In addition, non-use of tobacco as well as the need for family planning satisfied can also be considered effective coverage indicators as they capture the outcome of the intervention. The combination of tuberculosis detection and treatment success rates also provides information on effective coverage.

By contrast, for most service coverage indicators additional data collection and indicators are required to assess the quality of the implementation of the interventions. Additional indicators may describe health gains (e.g., survival rates on treatment), or service quality indicators, such as service provision (e.g., health worker adherence to protocols), or service readiness (e.g., availability of medicines or diagnostics). The OECD health care quality indicators provide examples of additional indicators used to measure quality [47].

## Targets

The definition of UHC implies that the ultimate goal is 100% coverage for all relevant interventions, which are added to the service and financial protection package according to country needs and possibilities. While 100% is a useful aspirational goal, empirical data suggest that this will only be possible for some coverage indicators.

Historic rates of progress in intervention coverage are available for several indicators related to the coverage of interventions for the health MDGs. Table 3 shows the year in which 80% and 95% coverage will be exceeded, on the basis of data from 33 low- and middle-income countries with two national surveys (Demographic and Health Survey [DHS] or Multiple Indicator Cluster Survey [MICS]); one survey was carried out in the 1990s, and one at least ten years later. The average survey interval is 12 years and the most recent survey was on average conducted in 2009. The computations are done for five indicators including antenatal care (at least one visit), skilled birth attendance, full immunization coverage among children 1 year old, family planning need satisfied, and a summary coverage index based on the unweighted mean of four maternal and child health intervention areas (maternity care, immunization, family planning, and treatment of sick children) described elsewhere [40]. Coverage of four antenatal visits is a preferred indicator over a single visit but data were not available from all surveys.

**Table 3:** Rate of progress and projected achievement dates for intervention coverage targets of 80% and 95% if past improvement rates continue at the same pace for the national level using past national progress rates; for the national level using the progress rate of the top progressor countries; for the poorest quintile using past poorest quintile progress rates: selected indicators in 33 countries with survey data in the mid-nineties and after 2005.

Population	Indicators	Median Coverage Rate Early Survey (1997)	Median Coverage Rate Recent Survey (2009)	Annual Rate of Increase in Coverage	Annual Rate of Reduction of Coverage Gap^a^	Year to Reach at Least 80%	Year to Reach at Least 95%
National progress continued	**ANC (1+)**	77.0	90.7	1.4	7.6	achieved^b^	2017
	**SBA**	47.3	61.0	2.1	2.5	2036	2091
	**Immunization**	47.7	63.2	2.4	2.9	2030	2077
	**FP**	56.4	66.5	1.4	2.2	2032	2095
	**Index**	56.6	68.4	1.6	2.7	2026	2078
National based on top progressors^c^	**ANC1**	47.5	84.0	4.8	10.0	achieved	achieved
	**SBA**	37.0	68.4	5.2	5.8	2021	2044
	**Immunization**	29.6	72.4	7.5	7.9	2017	2034
	**FP**	33.0	61.7	5.3	4.7	2020	2050
	**Index**	45.4	63.4	2.8	3.4	2023	2064
Poorest quintile	**ANC1**	59.8	82.8	2.7	7.1	achieved	2026
	**SBA**	22.2	34.2	3.6	1.4	2094	2192
	**Immunization**	33.5	50.6	3.5	2.5	2045	2101
	**FP**	35.5	57.2	4.0	3.5	2031	2071
	**Index**	42.1	58.2	2.7	2.7	2036	2086

a Coverage gap = 100−coverage (%).

b Achieved means achieved by 2015.

c Progress rate of top progressors (best quartile of countries) applied to national baseline rate.

ANC, at least one antenatal visit during pregnancy in the last three years; FP, need for family planning methods satisfied; Index, a summary measure based on eight indicators in four equally weighted intervention areas for maternal, newborn and child health (MNCH); SBA, skilled birth attendance; immunization, full coverage among children 12–23 months.

There are considerable differences between the indicators but some clear points emerge:

- Continuation of national progress rates during 1997–2009 is not adequate to reach 80% targets by 2030, the proposed target year for the post-2015 development agenda, for skilled birth attendance and family planning.
- Using the improvement rates of the best performing countries during 1997–2009 (top quartile of the 33 countries), national rates can easily achieve a target of 80% but not 95% by 2030.
- For the poorest quintile, current coverage improvement rates would be sufficient to achieve an 80% coverage target by 2030 only for one visit of antenatal care and almost for family planning but not for the other indicators.

There are also several measurement considerations that will have to be taken into account when setting targets. First, for many conditions, such as HIV, mental health, and surgical conditions, the exact population need will be hard to determine. Statistical modeling will be needed to obtain an estimate of the denominator but introduces considerable uncertainty in the coverage estimate. Therefore, targets of at least 80% or 90% are more useful for monitoring purposes.

Second, effective coverage rates of 100% are only possible if 100% effective treatments are available. Most treatments have considerable lower efficacy and effectiveness, making such high targets unachievable. Third, if summary measures are used, achievements tend to be lower. For instance, based on data from over 200 Demographic and Health Surveys, full immunization coverage rates (each child is fully immunized with all nationally recommended vaccines) tends to be almost one-fifth lower than the lowest individual vaccination coverage. A coverage index based on a mean of different individual intervention coverage rates (as used in Table 3), also tends to improve at a slower pace than some individual interventions.

Therefore, it is most useful to set targets based on empirical data using past baseline levels and trends, and taking into account measurement issues related to need, effective coverage, and summary measures. A global coverage target of a minimum of 80% in all populations is a useful general target for many interventions, though not all.

## Data Requirements

The two main data sources for intervention coverage indicators are household surveys and health facility reports. Household surveys should ideally collect information on the whole range of interventions, including indicators related to MDGs, NCDs, and injuries. In many low- and middle-income countries, household surveys, notably the Demographic and Health Surveys and UNICEF's Multiple Cluster Indicator Survey, have been the prime source of information for monitoring coverage of reproductive, maternal, and child health interventions related to the MDGs [48],[49]. Many high-income countries conduct regular health examination surveys but these are not yet commonplace in lower-income settings. A new generation of surveys should include a broader range of interventions, as most countries now face a wide spectrum of health challenges beyond those included in the MDGs. Health examination surveys with biological and clinical data collection, such as blood pressure, anthropometry, vision, and serology are needed. A comprehensive health examination survey at least once every five years using internationally accepted standards of data collection and analysis would provide a wealth of information for UHC monitoring. In countries experiencing rapid epidemiological change, an intermediate survey is justified.

Health facility reports provide data on the numerators for coverage indicators, such as immunization, deliveries, and antiretroviral therapy use. They should include the private sector, which is often a problem, and also data provided through community delivery strategies. The advantage of health facility data is that they can be used for annual monitoring at national and subnational levels, but data quality is often poorer than for surveys, private sector reporting may be limited, and denominators need to be estimated [50]. The latter are generally obtained from population census projections, which may have considerable error because of changing growth and migration rates. However, efforts to improve routine health facility data quality, for example by improving timeliness and accuracy of reporting through web-based systems [51], can pay off in terms of the availability of data on coverage. For several treatment indicators, such as TB or HIV, facility statistics are often the only source. For other coverage indicators, both survey and health facility data are available, providing an opportunity to evaluate data quality and make adjustments where needed.

Moving towards UHC is not only a matter of improving average levels but also about reducing disparities and improving equity. Therefore, indicator disaggregation should be possible by sex, age, household wealth/income, gender, residence (urban/rural, province, district), and other stratifiers [52]. Population-based surveys usually include a range of stratifiers, which permit multiple disaggregations. The main drawback from surveys is that sample sizes are often too small for detailed subnational disaggregation. Data collected from health facilities or administrative reports allow subnational disaggregation by geographic area, and can be collected on a continuous basis. The level of disaggregation by age and sex is however limited, unless there is an individual level electronic medical records system in place.

## Discussion

A few measurable and understandable indicators to monitor progress can be a powerful way of galvanizing efforts to move towards UHC. Countries should not limit themselves to tracking a small set of indicators that are included in internationally agreed development goals but should also work to progressively include additional indicators that are locally important. Because countries face very diverse circumstances in terms of epidemiology, health systems, and financing, levels of socioeconomic development and population demands, priority interventions, and associated indicators are likely to vary by place and over time, related to the progressive realization of the UHC goal. Therefore, the meaning and implementation of UHC may also vary, and affect the ways in which progress is measured. It is however possible to identify a set of global tracer indicators that all countries should monitor.

First and foremost, UHC monitoring needs to be embedded in overall health progress and system performance assessment in countries. Progress towards UHC requires strengthening health sector inputs, service delivery and quality, and access. The logical results chain commonly used in monitoring of national health sector strategies provides a framework for identifying and measuring the most important factors and informing the policy dialogue on UHC [4]. There is also a need to monitor the social and environmental determinants of health, as these have an important impact, along with UHC, on health. The papers presented in this PLOS Collection on UHC monitoring provide useful illustrations of how the framework can be used in the country context. Box 3 summarizes the recommended steps to incorporate UHC monitoring in countries.

**Box 3. Recommended Steps for Country Monitoring of UHC**

- Ensure there is a fully developed regular system of health progress reviews and systems performance assessment of the national health sector strategic plan, including annual health sector reviews;
- Embed UHC monitoring within the overall monitoring and review system;
- Select a set of tracer indicators for financial protection and coverage, divided into promotion and prevention, and treatment, that address the main intervention areas;
- Ensure special attention for the quality dimension of the interventions, either within the same indicator or through additional indicators;
- Set targets for the intervention coverage indicators based on past trends, new investments, and international targets, ensuring consistency with health impact goals, nationally and for the most disadvantaged population such as the poorest quintile, those living in poorer areas, gender, and other country-relevant stratifiers;
- Consider additional monitoring of general service utilization by wealth quintile and other relevant stratifiers, taking into account underreporting of need for services by the more disadvantaged populations;
- Conduct regular progress assessments as part of reviews of the implementation of the health sector strategic plan, with a focus on the progress among the most disadvantaged populations.
- Invest in data sources that should include timely, accurate, complete facility data, and a regular health examination survey that collects information on all priority health topics.

Within the context of comprehensive monitoring of health sector progress and performance, we propose that UHC monitoring focus on intervention coverage indicators [2]. In addition to financial protection, intervention coverage with quality services is the most direct result of efforts to enhance access and quality of services to the population. Improvements in coverage among the most disadvantaged populations, such as the poorest individuals, are essential for progress towards UHC.

Globally, there are many candidate service coverage indicators, which can be classified in different ways. A global measure of progress can only be synthesized from country data if there is a common and comparable set of tracer indicators that meet international measurement standards. Consequently, all countries would commit to monitoring a small set of core indicators on a regular basis using global measurement standards, allowing a “roll-up” of the country data into global monitoring. In this paper, we illustrated this type of data with a dozen prevention and treatment coverage indicators.

A common measure relevant to UHC monitoring everywhere, regardless of the level of socioeconomic development or epidemiological context, has drawbacks. Monitoring a concept as complex and multifaceted as UHC inevitably involves making decisions about what elements should be included and whether it is desirable or possible to bring them together in a summary measure. The identification, through a reductionist approach, of a few tracer indicators may have unintended consequences that arise with all indicator constructs, including greater investment in the interventions selected for monitoring than in non-selected interventions (“gaming”). In addition, the association between the tracer indicators and overall progress in intervention coverage may not be particularly strong and might change over time.

In principle, coverage indicators should have a 100% target. In practice, only a few interventions reach such high coverage rates nationally and in all population groups, and effective coverage measures tend to fall far short on this. While maintaining universal intervention coverage as the goal, countries should establish their own benchmarks depending on baseline and the feasibility of achieving progress, with sufficient balance between aspiration and realism. A general target for intervention coverage could be a minimum of 80% coverage among the poorest or any other disadvantaged population.

The process of identification of good tracer indicators shows that there are major gaps. First, there is a lack of measurable coverage indicators for several health priorities such as mental health issues, injuries, disability, and others. Second, most treatment indicators do not have reliable denominators, as population need is difficult to measure, especially for treatment interventions for which potentially high out-of-pocket expenses are most likely a limiting factor to service use. While the size of the population in need of services decreases, when moving from health promotion and prevention to tertiary-level care, the service costs and financials for individuals and families may increase dramatically, owing to specialist interventions and medicines. Third, only a few indicators have a dimension that captures the quality of services. Most indicators need supplementary indicators on quality of service delivery or health impact. Further research and investment are needed to address these gaps, which should be a priority for research in the coming years [53].

Monitoring progress towards UHC will build upon previous experiences, for example in monitoring progress towards the MDGs, but must necessarily expand beyond these to adopt new domains of health action and incorporate innovative metrics, methodologies, and measurement techniques. The implications for country information systems are profound, necessitating sustained support from the research, statistical, and development community.

## Supporting Information

Table S1 **Assessment of indicators using standard criteria.**
(DOCX)

## Abbreviations

MDG: Millennium Development Goal
NCD: noncommunicable disease
UHC: universal health coverage

## References

[1] World Health Organization (2010) The world health report - health systems financing: the path to universal coverage. Geneva: World Health Organization. Available: http://www.who.int/whr/en/index.html. Accessed 14 December 2013. 10.2471/BLT.10.078741 PMC2878164 20539847

[2] Boerma T, Eozenou P, Evans D, Evans T, Kieny M-P, et al (2014) Monitoring progress towards universal health coverage at country and global levels. PLoS Med 11: e1001731 10.1371/journal.pmed.1001731 25243899 PMC4171369

[3] Saksena P, Hsu J, Evans D (2014) Financial risk protection and universal health coverage: evidence and measurement challenges. PLoS Med 11: e1001701 10.1371/journal.pmed.1001701 25244520 PMC4171370

[4] Bertrand JT, Magnani RJ, Rutenberg N (1996) Evaluating family planning programs: with adaptations for reproductive health. University of North Carolina, Chapel Hill: The Evaluation Project.

[5] Boerma T, Pisani E, Schwartländer B, Mertens T (2000) A framework for the evaluation of national AIDS programmes. MEASURE Evaluation WP-99-17. Chapel Hill, North Carolina: The Evaluation Project.

[6] Bryce J, Victora CG, Boerma T, Peters DH, Black RE (2011) Evaluating the scale-up for maternal and child survival: a common framework. Int Health 3: 139–146. 24038362 10.1016/j.inhe.2011.04.003

[7] Bryce J, Victora CG, Habicht JP, Black RE, Scherpbier RW (2005) MCE-IMCI Technical Advisors (2005) Programmatic pathways to child survival: results of a multi-country evaluation of Integrated Management of Childhood Illness. Health Policy Plan 20 Suppl 1: i5–i17. 16306070 10.1093/heapol/czi055

[8] World Health Organization (2011) Monitoring, evaluation and review of national health strategies: a country-led platform for information and accountability. International Health Partnership+ and WHO, Geneva. Available: http://www.who.int/healthinfo/country_monitoring_evaluation/documentation/en/index.html. Accessed 21 September 2013.

[9] Evans DB, Hsu J, Boerma T (2013) Universal health coverage and universal access. Bull World Health Organ 91: 546–546A. 23940398 10.2471/BLT.13.125450 PMC3738317

[10] Tanahashi T (1978) Health service coverage and its evaluation. Bull World Health Organ 56: 295–303. 96953 PMC2395571

[11] Penchansky R, Thomas JW (1981) The concept of access: definition and relationship to consumer satisfaction. Med Care 19: 127–140. 7206846 10.1097/00005650-198102000-00001

[12] Shengelia B, Tandon A, Adams OB, Murray CJL (2005) Access, utilization, quality, and effective coverage: an integrated conceptual framework and measurement strategy. Soc Sci Med 61: 97–109. 15847965 10.1016/j.socscimed.2004.11.055

[13] United Nations Statistics Division (2008) Official list of MDG indicators. New York: United Nations. Available: http://mdgs.un.org/unsd/mdg/host.aspx?Content=indicators/officiallist.htm. Accessed 14 December 2013.

[14] Requejo JH, Newby H, Bryce J (2013) Measuring coverage in MNCH: challenges and opportunities in the selection of coverage indicators for global monitoring. PLoS Med 10: e1001416. 23667336 10.1371/journal.pmed.1001416 PMC3646210

[15] World Health Assembly (2013). Resolution EB 132/7. Draft action plan for the prevention and control of noncommunicable diseases 2013–2020. Geneva: World Health Assembly.

[16] WHO, Countdown to 2015, Health Metrics Network and UNICEF (2011) Monitoring maternal, newborn and child health: understanding key progress indicators. Geneva: World Health Organization.

[17] Beckett M, Weinstein M, Goldman N, Yu-Hsuan L (2000) Do health interview surveys yield reliable data on chronic illness among older respondents? Am J Epidemiol 151: 315–323. 10670557 10.1093/oxfordjournals.aje.a010208

[18] World Health Organization (2013) Global status report on road safety 2013: supporting a decade of action. Geneva: World Health Organization.

[19] United Nations Economic and Social Commission for Asia and the Pacific (UNESCAP) (2012) Incheon strategy to “make the right real” for persons with disabilities in Asia and the Pacific. Bangkok Available: http://www.unescap.org/resources/incheon-strategy-%E2%80%9Cmake-right-real%E2%80%9D-persons-disabilities-asia-and-pacific. Accessed 14 December 2013.

[20] World Health Organization and United Nations Economic and Social Commission for Asia and the Pacific (UNESCAP) (2008) Training manual on disability statistics. Bangkok Available: http://www.cbm.org/article/downloads/82788/Training_Manual_Disability_Statistics_WHO.pdf. Accessed 14 December 2013.

[21] Cornman JC, Freedman VA, Agree EM (2005) Measurement of assistive device use: implications for estimates of device use and disability in late life. Gerontologist 45: 347–358. 15933275 10.1093/geront/45.3.347

[22] Bryce J, Arnold F, Blanc A, Hancioglu A, Newby H, et al (2013) Measuring coverage in MNCH: new findings, new strategies, and recommendations for action. PLoS Med 10: e1001423. 23667340 10.1371/journal.pmed.1001423 PMC3646206

[23] Hosseinpoor AR, Bergen N, Mendis S, Harper S, Verdes E, et al (2012) Socioeconomic inequality in the prevalence of noncommunicable diseases in low- and middle-income countries: results from the World Health Survey. BMC Public Health 12: 474. 22726343 10.1186/1471-2458-12-474 PMC3490890

[24] Levesque JF, Mukherjee S, Grimard D, Boivin A, Mishra S (2013) Measuring the prevalence of chronic diseases using population surveys by pooling self-reported symptoms, diagnosis and treatments: results from the World Health Survey of 2003 for South Asia. Int J Public Health 58: 435–447. 23436012 10.1007/s00038-013-0446-5

[25] Kowal P, Chatterji S, Naidoo N, Biritwum R, Fan W, et al (2012) Data resource profile: the World Health Organization Study on global AGEing and adult health (SAGE). Int J Epidemiol 41: 1639–1649. 23283715 10.1093/ije/dys210 PMC3535754

[26] WHO World Mental Health Survey Consortium (2004) Prevalence, severity, and unmet need for treatment of mental disorders in the World Health Organization World Mental Health Surveys. JAMA 291: 2581–2590. 15173149 10.1001/jama.291.21.2581

[27] Groen RS, Samai M, Stewart KA, Cassidy LD, Kamara TB, et al (2012) Untreated surgical conditions in Sierra Leone: a cluster randomized, cross-sectional, countrywide survey. Lancet 380: 1082–1087. 22898076 10.1016/S0140-6736(12)61081-2

[28] WHO (2011) Use of glycated haemoglobin (HbA_1c_) in the diagnosis of diabetes mellitus. Available: www.who.int/diabetes/publications/report-hba1c_2011.pdf. Accessed 14 December 2013.

[29] Bourne RR, Rosser DA, Sukudom P, Dineen B, Laidlaw DA, et al (2003) Evaluating a new logMAR chart designed to improve visual acuity assessment in population-based surveys. Eye (Lond) 17: 754–758. 12928690 10.1038/sj.eye.6700500

[30] National Research Council (US) Committee on Population; Finch CE, Vaupel JW, Kinsella K, editors (2001). Cells and surveys: should biological measures be included in social science research? Washington (D.C.): National Academies Press (US). 23166967

[31] Mahy M, Tassie JM, Ghys P, Stover J, Beusenberg M, et al (2010) Estimation of antiretroviral therapy coverage: methodology and trends. Curr Opinion HIV AIDS 5: 97–102. 10.1097/COH.0b013e328333b892 20046154

[32] WHO (2013) Global Tuberculosis report 2013. Geneva: World Health Organization.

[33] Yates R (2009) Universal health care and the removal of user fees. Lancet 373: 2078–2081. 19362359 10.1016/S0140-6736(09)60258-0

[34] Organization for Economic Development (OECD) (2013), Health at a Glance 2013: OECD Indicators, OECD Publishing. Available: 10.1787/health_glance-2013-en. Accessed 13 December 2013. Accessed 14 December 2013.

[35] Aguilera X, Castillo-Laborde C, Nájera-De Ferrari M, Delgado I, Ibañez C (2014) Monitoring and evaluating progress towards universal health coverage in Chile. PLoS Med 11: e1001676 10.1371/journal.pmed.1001676 25244581 PMC4171380

[36] de Looper M, Lafortune G (2009) Measuring disparities in health status in access and use of health care in OECD countries. OECD Health Working Papers No. 43. Paris: OECD.

[37] Meng Q, Xu L, Zhang Y, Qian J, Cai M, et al (2012) Trends in access to health services and financial protection in China between 2003 and 2011: a cross-sectional study. Lancet 379: 805–814. 22386034 10.1016/S0140-6736(12)60278-5

[38] Bhandari A, Wagner T (2006) Self-reported utilization of health care services: improving measurement and accuracy. Med Care Research and Reviews 63: 217–235. 10.1177/1077558705285298 16595412

[39] Devaux M, de Looper M (2012), “Income-Related Inequalities in Health Service Utilisation in 19 OECD Countries, 2008–2009”, *OECD Health Working Papers* 58. Paris: OECD Publishing. Available: 10.1787/5k95xd6stnxt-en.

[40] Boerma JT, Bryce J, Kinfu Y, Axelson H, Victora CG (2008) Mind the gap: equity and trends in coverage of maternal, newborn and child health services in 54 Countdown countries. Lancet 371: 1259–1267. 18406860 10.1016/S0140-6736(08)60560-7

[41] Victora CG, Barros AJ, Axelson H, Bhutta ZA, Chopra M, et al (2012) How changes in coverage affect equity in maternal and child health interventions in 35 Countdown to 2015 countries: an analysis of national surveys. Lancet 380: 1149–1156. 22999433 10.1016/S0140-6736(12)61427-5

[42] Kumar C, Kumar Singh P, Kumar Rai R (2013) Coverage gap in maternal and child health services in India: assessing trends and regional deprivation during 1992–2006. J Public Health 35: 598–606. 10.1093/pubmed/fds108 23359666

[43] World Health Organization (2013). Global Health Observatory. Database. Available: http://www.who.int/gho/database/en/. Accessed 23 December 2013.

[44] The DHS Program (2013) Available: http://www.measuredhs.com/ (accessed 23 December 2013).

[45] World Health Organization (2013). STEPwise approach to surveillance. Available: http://www.who.int/chp/steps/en/. Accessed 23 December 2013.

[46] Huda T, Khan JAM, Ahsan KZ, Jamil K, El Arifeen S (2014) Monitoring and evaluating progress towards universal health coverage in Bangladesh. PLoS Med 11 9: e1001722. 25244599 10.1371/journal.pmed.1001722 PMC4170958

[47] Kelly E, Hurst J (2006). Health care quality indicators project: conceptual framework paper. Organization for Economic Cooperation and Development. Working papers 23, Paris. Available: http://www.oecd.org/els/health-systems/36262363.pdf.

[48] Bhutta Z, Chopra M, Axelson H, Berman O, Boerma T, et al (2010) Countdown to 2015 decade report (2000–2010): taking stock in progress in maternal, newborn and child survival. Lancet 375: 2032–2044. 20569843 10.1016/S0140-6736(10)60678-2

[49] WHO and Health Metrics Network (2011) Country health information systems. A review of the current situation and trends. Geneva: WHO. Available: http://www.who.int/healthmetrics/news/chis_report.pdf?ua=1.

[50] Abouzahr C, Boerma T (2005) Health information systems: the foundation of public health. Bull World Health Organ 83: 578–583. 16184276 PMC2626318

[51] Braa J, Heywood A, Sahay S (2012) Improving quality and use of data through data-use workshops: Zanzibar, United Republic of Tanzania. Bull World Health Organ 90: 379–384. 22589572 10.2471/BLT.11.099580 PMC3341693

[52] Hosseinpoor AR, Bergen N, Koller T, Prasad A, Schlotheuber A, et al (2014) Equity-oriented monitoring in the context of universal health coverage. PLoS Med 11 9: e1001727 10.1371/journal.pmed.1001727 25243463 PMC4171107

[53] World Health Organization (2013) Research for universal health coverage. World Health Report. Geneva: World Health Organization.

