# Supplementary material for: Monitoring Intervention Coverage in the Context of Universal Health Coverage
Source: PLoS Med. 2014 Sep 22;11(9):e1001728. doi: 10.1371/journal.pmed.1001728 (PMC4171108; doi:10.1371/journal.pmed.1001728)
Supplement: Table S1 — Assessment of indicators using standard criteria. (DOCX) [file pmed.1001728.s001.docx]

| **WEB TABLE 1: ASSESSMENT OF INDICATORS USING STANDARD CRITERIA** | | | | | |  |  |  |  |  |  |  |
| --- | --- | --- | --- | --- | --- | --- | --- | --- | --- | --- | --- | --- |
| **Health condition** | **Intervention area** | **Indicator name** | **Clear target** | **Epidemio-logical relevance** | **Proven effective intervention** | **Measurability** | **Easy to commu-nicate** | **Equity** | **Data availability** | **Main data source** | **Initiative** | **Comment** |
| **Unintended pregnancies** | Family planning | Need for family planning satisfied | Yes, 100% | High | Yes | Good, surveys, but requires multiple questions; | Yes | Yes, all stratifiers | Good | Survey | MDG 5b | Can use contraceptive prevalence rate as proxy; |
| **Antenatal care** | Pregnancy care | Antenatal care, 4 or more visits | Yes, 100% | High | Yes, but quality is problem | Good, surveys or facility reports | Yes | Yes, all stratifiers | Good | Survey + facility data | MDG 5b |  |
| **Antenatal care / HIV** | Pregnancy care (HIV) | HIV positive pregnant women receving ARVs for prophylaxis | Yes, 100% | High, if HIV common | Yes | Moderate, facility data | Yes | Yes, all stratifiers | Fair | Facility data | UNGASS |  |
| **Antenatal care / malaria** | Pregnancy care (malaria) | At least 2 doses of SP during pregnancy (IPTp) | Yes, 100% | High if malaria endemic | Yes | Good, survey, recall by women | Yes | Yes, all stratifiers | Good | Surveys + facility data |  |  |
| **Maternal and newborn health issues** | Maternal and newborn care | Skilled birth attendance | Yes, 100% | High | Yes | Good, surveys | Yes | Yes, all stratifiers | Very good | Survey + facility data | MDG 5a | Can use institutional delivery rate as proxy |
|  | Postpartum care | Postnatal care visit | Yes, 100% | High | Yes, if quality of care is good | Good, surveys or facility reports | Yes | Yes, all stratifiers | Fair | Survey + facility data | COIA |  |
| **Childhood illnesses** | Vaccination | DTP3/ pentavalent vaccine | Yes, 100% | High | Yes | Very good; surveys and facility reports | Yes | Yes, all stratifiers | Very good | Survey + facility data | MDG | Coverage of other vaccines (measles, BCG, polio etc.) also good candidates |
|  | Treatment of sick children | Sick child with ARI symptoms taken for assessment to clinic | Yes, 100% | High | Yes, for pneumonia if treated | Fair, but denominator (need) issues | Yes | Yes, if numbers are adequate | Fair | Survey | COIA |  |
|  |  | Sick child with diarrhoea receiving ORT / ORS | Yes | High | Yes | Fair, denominator issues | Yes | Yes, if numbers are adequate | Good | Survey |  | ORS use also possible for diarrhoea but target may not be 100% |
| **Malaria** | Malaria prevention | Household ownership of ITN | Yes, 100% | High, in endemic areas | Yes | Good, surveys only | Yes | Yes, all stratifiers | Good | Survey | MDG 6 | ITN use also possible |
|  | Treatment | Child with fever taken to clinic | Yes, 100% | High, in endemic areas | Yes, if followed by treatment | Fair | Yes | Yes | Good | Survey | MDG 6 |  |
| **Tuberculosis** | TB treatment | TB treatment | Yes, 100% | High | Yes | Good for treatment outcome, denominator estimated; facility data only | Yes | Partially, sex, age, location | Good | Facility data | MDG 6 | Combines detection rate and treatment success rate |
| **HIV/AIDS** | Prevention | Condom use with higher risk partner | Yes, 100% | High | Yes | Moderate, reporting bias in surveys | Fair | Yes, all stratifiers | Fair | Survey | MDG 6 | Better to combine condom use with prevalence of high risk behaviour |
|  | HIV therapy | ARV treatment | Yes, 100% | High | Yes | Moderate, facility data, denominator estimation | Fair | Partially, sex, age, location | Fair | Facility data | MDG 6 |  |
| **Environmental health problems** | Water and sanitation | Safe water source | Yes, 100% | High | Yes | Good | Yes | Yes, urban-rural or wealth | Good | Survey | MDG 7 |  |
|  | Water and sanitation | Adequate sanitation | Yes, 100% | High | Yes | Good | Yes | Yes, urban-rural or wealth | Good | Survey | MDG 7 |  |
|  | Air quality | No indoor use of solid fuels | Yes, 100% | High | Yes | Fair | Yes | Yes, urban-rural or wealth | Fair | Survey | MDG 7 |  |
|  | Air quality | People living in cities with poor air quality | Yes | High | Yes | Fair | Yes | No | Fair | Surveillance |  | Needs standardi-zation using PM 2.5, continuous measurement |
| **Nutritional deficiencies** | Breast-feeding | Children ever breastfed | Yes, 100% | Fair, in high mortality settings | Yes | Good | Yes | Yes | Good | Survey |  |  |
|  |  | Children exclusively breasfed 0-5 months | Fair | High, in high mortality settings | Yes | Fair, based on current status data, which limits sample size | Yes | Poor, because of sample size | Good for aggregate | Survey |  |  |
|  | Micro-nutritients | Households with iodized salt | Yes, 100% | Yes | Yes | Good | Yes | Yes | Fair, requires salt sample | Survey |  |  |
|  |  | Vitamin A supple-mentation in children | No, only in low income settings | High in high mortality settings | Yes | Good | Yes | Yes | Good | Survey |  |  |
| **Cardiovascular diseases** | Promotion (behaviour) | Salt intake per person per day | Yes, < 5g per day | High | Yes | Requires 24hr urinary output in surveys | Yes | Yes, all stratifiers | Poor | Survey | NCD monitoring plan |  |
|  | Promotion (behaviour) | Obesity/ overweight prevalence | Yes | High | Limited success | Good, anthropometry | Yes | Yes | Good | Surveys | NCD monitoring plan |  |
|  | Promotion (behaviour) | Adequate physical activity | Yes, per week | High | Yes | Special modules in surveys | Yes | Yes | Fair | Surveys | NCD monitoring plan |  |
|  | Prevention | Hypertension treatment | Yes, 100% | High | Yes | Good, requires measurement blood pressure in surveys | Yes | Yes, all stratifiers | Fair | Survey | NCD monitoring plan | Is effective treatment if treatment status is taken into account |
|  |  | Normal energy intake from saturated fatty acids | Yes, 100% | High | Yes | Fair, dietary recall | Yes | Yes | Poor | Survey | NCD monitoring plan |  |
|  | Prevention | Normal cholesterol | Yes, 100% | High | Yes | Good, requires blood sample | Yes | Yes, all stratifiers | Fair | Survey | NCD monitoring plan |  |
|  | Prevention | Multi-drug treatment of people at higher risk of CVD | Yes, 100% | High | Yes | Fair, requires risk assessment for need | Fair | Yes, all stratifiers, if measured in surveys | Poor | Survey | NCD monitoring plan | Requires multiple questions to assess risk |
| **Cancer** | Prevention | Non use of tobacco | Yes, 100% | High | Yes | Good | Yes | Yes | Good | Survey | NCD monitoring plan |  |
| **Liver / pancreas conditions (traffic injuries)** | Prevention | No harmful use of alcohol | Yes, 100% | High | Yes | Poor | Yes | Yes | Poor | Survey | NCD monitoring plan |  |
|  | Prevention / early treatment | Cervical cancer screening | Yes | High | Yes | Good | Yes | Yes | Poor | Survey + facility data | NCD monitoring plan | Mammography is another candidate, more discussion on population benefits |
|  | Prevention | Hepatitis B vaccination | Yes, 100% | High | Yes | Good | Yes | Yes | Good | Survey + facility data | NCD monitoring plan |  |
|  | Prevention | HPV vaccination (adolescent girls) | Yes, 100% | High | Yes | Good | Yes | Yes | Poor | Survey + facility data |  | Cervical cancer screening is an alternative |
| **Other chronic adult conditions** | Treatment | Chronic condition treatment (arthritis, asthma, angina, chronic respiratory disease) | Yes, 100% | Mostly High (variable by condition) | Partial | Fair | Yes | Yes | Poor | Survey |  | Based on survey questions and self-reported diagnosis |
|  | Treatment | Depression treatment | Yes, 100% | High | Yes | Fair | Yes | Yes | Fair | Survey |  | Based on survey questions and self-reported diagnosis |
|  | Treatment | Adequate vision | Yes, 100% | High | Yes | Good, requires vision measurement in surveys | Yes | Yes | Fair | Survey |  | Effective treatment if treatment status is taken into account |
|  | Treatment | Normal blood glucose levels | Yes, 100% | High | Yes | Good, with serological test | Yes | Yes | Poor | Survey | NCD monitoring plan | Effective treatment if treatment status is taken into account |
|  | Treatment | Dental care visit | Yes, 100% | Moderate | Yes | Fair, based on self reported need | Yes | Yes | Poor | Survey |  | Is more a utilization figure, unless is based on regularity of visits |
| **Injuries and surgical conditions** | Treatment | Injury treatment | Yes, 100% | High | Yes | Fair, based on self reported need | Yes | Yes | Poor | Survey |  |  |
|  | Prevention | Helmet use | Yes | High | Yes | Good | Yes | No | Fair | Police surveys |  | Policies could be used as proxy |
|  |  | Seatbelt use | Yes | High | Yes | Good | Yes | No | Fair | Police surveys |  | Policies could be used as proxy |
|  | Surgical care | Surgical assessment | Yes, 100% | Moderate | Yes | Fair, based on self reported need | Yes | Yes | Poor | Survey |  | SOSAS instrument, focus on self-reported condition |
|  |  |  |  |  |  |  |  |  |  |  |  |  |
